# A Novel Roseosiphophage Isolated from the Oligotrophic South China Sea

**DOI:** 10.3390/v9050109

**Published:** 2017-05-15

**Authors:** Yunlan Yang, Lanlan Cai, Ruijie Ma, Yongle Xu, Yigang Tong, Yong Huang, Nianzhi Jiao, Rui Zhang

**Affiliations:** 1 State Key Laboratory of Marine Environmental Science, Institute of Marine Microbes and Ecospheres, Xiamen University (Xiang’an), Xiamen 361102, Fujian, China; yangyunlan@stu.xmu.edu.cn (Y.Y.); cailanlan@stu.xmu.edu.cn (L.C.); maruijie@stu.xmu.edu.cn (R.M.); xuyongle3884778@xmu.edu.cn (Y.X.); 2 Beijing Institute of Microbiology and Epidemiology, State Key Laboratory of Pathogen and Biosecurity, Beijing 100071, China; tong.yigang@gmail.com (Y.T.); presidenthuang@126.com (Y.H.)

**Keywords:** roseophage, genome, phylogenetic analysis, environmental distribution

## Abstract

The *Roseobacter* clade is abundant and widespread in marine environments and plays an important role in oceanic biogeochemical cycling. In this present study, a lytic siphophage (labeled vB_DshS-R5C) infecting the strain type of *Dinoroseobacter shibae* named DFL12^T^, which is part of the *Roseobacter* clade, was isolated from the oligotrophic South China Sea. Phage R5C showed a narrow host range, short latent period and low burst size. The genome length of phage R5C was 77, 874 bp with a G+C content of 61.5%. Genomic comparisons detected no genome matches in the GenBank database and phylogenetic analysis based on DNA polymerase I revealed phylogenetic features that were distinct to other phages, suggesting the novelty of R5C. Several auxiliary metabolic genes (e.g., *phoH* gene, heat shock protein and queuosine biosynthesis genes) were identified in the R5C genome that may be beneficial to the host and/or offer a competitive advantage for the phage. Among siphophages infecting the *Roseobacter* clade (roseosiphophages), four gene transfer agent-like genes were commonly located with close proximity to structural genes, suggesting that their function may be related to the tail of siphoviruses. The isolation and characterization of R5C demonstrated the high genomic and physiological diversity of roseophages as well as improved our understanding of host–phage interactions and the ecology of the marine *Roseobacter*.

## 1. Introduction

As the most abundant biological entities, viruses play an important role in nutrient cycles and energy flow in marine environments through viral lysis [1]. Viruses are also one of the major contributors to horizontal gene transfer and evolution of their hosts, with approximately 10^23^ infections occurring every second in seawater [2]. Recent investigations of viruses using metagenomics fundamentally changed our estimation of their diversity and community structure as well as our understanding of their interaction with their hosts [3]. Despite the tremendous amount of genetic information provided by virome studies, most are considered “dark material” owing to the lack of similarity to known sequences. It is proposed and demonstrated that this problem can be partially solved by the isolation and genetic characterization of viruses, especially those that infect dominant bacterial groups, such as *Synechococcus* and *Vibrio* in coastal areas, in addition to SAR 11 and *Prochlorococcus* in open ocean [4,5,6,7,8]. In addition, the physiological and ecological characterization of these isolated viruses, as well as their interaction with hosts, has improved our understanding of their ecological and biogeochemical roles in real environments [9,10].

In recent years, increasing attention has been paid to *Roseobacter* and their phages because of their worldwide distribution, high abundance, and possible ability to be cultured [11,12,13,14,15,16]. It is estimated that the *Roseobacter* clade contributes up to 15–20% of the total bacterioplankton in typical coastal areas and open oceans [17]. They are detected from a variety of marine habitats ranging from marine snow, micro and macro algae, microbial mats, sediments, sea ice, and hydrothermal vents [11]. Several major biogeochemical processes, such as the transformation of organic and inorganic sulfur compounds, carbon monoxide oxidation, and the degradation of high molecular weight organic matter, are mediated by *Roseobacter* [11,17]. In addition, they are easily isolated and fast-growing, which is advantageous for phage studies compared with other numerically dominant marine bacteria, such as SAR11 and *Prochlorococcus*.

The genus *Dinoroseobacter* is one of the most well-studied groups of marine bacteria [11,18]. *Dinoroseobacter shibae*, the type species of *Dinoroseobacter*, was isolated from the dinoflagellate *Prorocentrum lima* and lives in a symbiotic relationship with marine algae [19]. *D. shibae* is an aerobic anoxygenic phototrophic bacterium and is competitive in unpredictable, changing environments, because of its ability to perform light-driven ATP synthesis and its novel acylated homoserine lacton compounds [20,21,22,23]. The strain type *D. shibae* DFL12^T^ has been completely sequenced and exhibits complex viral defense systems (i.e., clustered regularly interspaced small palindromic repeats, CRISPRs) encoded in its genome [19,24].

To date, 17 phages and prophages infecting *Roseobacter* have been reported [12,13,14,16,25,26,27,28,29,30,31,32]. It has been shown that roseophages contain a considerable degree of genomic variability [29,33]. However, compared with the biogeographical, physiological and ecological diversity of *Roseobacter*, the range of roseophages studied so far remains relatively narrow. For example, most roseophages were isolated from coastal areas, but *Roseobacter* was also shown to be widely distributed in open ocean [13,29]. Viral metagenomic recruitments based on available roseophage genome sequences indicated the presence of roseophages in open ocean, but only one such organism has been isolated from this environment [34]. In addition, most of the roseophages belong to the *Podoviridae* family, but previous studies of dominating marine bacteria groups (e.g., *Synechococcus* and *Prochlorococcus*) have suggested that they are also infected by *Siphoviridae* or *Myoviridae* family phages. Phages adapted to different environments and with different phylogenetic properties are driving factors for ecological distribution and behavior of their hosts. Therefore, the aim of this study was to isolate and characterize more phages for *Roseobacter*, particularly *D. shibae*, from open ocean areas, such as the oligotrophic South China Sea. We expect that the phages isolated from distinct geographical environments and phylogenetic families will provide novel information regarding their genetics, diversity and distribution, which will expand our knowledge on the viral ecology of the marine *Roseobacter*.

## 2. Materials and Methods

### 2.1. Isolation and Purification of Phages

*D. shibae* DFL12^T^ was used as the host in this study and was maintained on RO medium (yeast extract 1 g·L^−1^, peptone 1 g·L^−1^, sodium acetate 1 g·L^−1^, artificial seawater 1 L, pH 7.4–7.8) at room temperature. To increase the probability of phage isolation, viruses in the surface water of the South China Sea, which was characterized as an oligotrophic environment, were enriched by tangential flow filtration with a 30 kDa polysulfone cartridge (Labscale, Millipore, CA, USA). Double-layer agar was used for the isolation and purification of phages.

After purifying five times, phages were cultivated for expansion in liquid RO medium. The culture was centrifuged at 12,000× *g* at 4 °C for 10 min to obtain the phage-containing supernatant. Following this, phages were concentrated by precipitation with polyethylene glycol 8000 (final concentration: 100 g·L^−^^1^) and centrifuged at 10,000× *g* for 60 min at 4 °C. The precipitate was resuspended in SM buffer (100 mM NaCl, 8 mM MgSO_4_, 50 mM Tris-HCl at pH 7.5) and purified by CsCl equilibrium gradient centrifugation (200,000× g, 4 °C, 24 h). The pellet was dialyzed through 30 kDa super-filters (UFC503096, Millipore) and the filtrate was collected for morphologic observation and DNA extraction.

### 2.2. Transmission Electron Microscopy (TEM)

The purified and desalted phages were diluted to the appropriate concentration with SM buffer and adsorbed onto 200-mesh carbon-coated coppers for 10–30 min in the dark. After being stained with 1% phosphotungstic acid and dried for 30 min, samples were viewed at 80 kV voltage using a JEM-2100 transmission electron microscope (JEOL, Tokyo, Japan). Images were collected using the CCD image transmission system (Gatan Inc., Pleasanton, CA, USA).

### 2.3. Host Range

Besides *D. shibae* DFL12^T^, bacterial strains used in the host range test included *Citromicrobium* sp. WPS32, *Citromicrobium* sp. JL2201, *Citromicrobium* sp. JL1351, *Citromicrobium* sp. JL354, *Citromicrobium* sp. JL1363, *Dinoroseobacter* sp. JL1447, *Erythrobacter litoralis* DMS 8509, *Erythrobacter longus* DMS 6997, *Erythrobacter* sp. JL475, *Hoeflea phototrophica* DFL-43, *Paenibacillus* sp. JL1210, *Roseobacter denitrificans* OCh114, *Roseomonas* sp. JL2290, *Roseomonas* sp. JL2293, *Roseovarius* sp. JL2434, *Ruegeria* sp. JL126, *Spingobium* sp. JL1088 and *Silicibacter pomeroyi* DSS3. Exponentially growing cultures of these bacteria strains were incubated with phages, which were diluted to 10^2^, 10^4^, 10^6^, and 10^8^ PFU mL^−1^, for 20 min and then plated using a plaque assay. Replication was conducted for each bacterium strain.

### 2.4. One-Step Growth Curve

To analyze the infectivity and replication ability of phages, a one-step growth curve test was performed. Phages were added to 1 mL of log-phase *D. shibae* DFL12^T^ with a multiplicity of infection of 0.01, before being incubated for 25 min at room temperature in the dark. The culture was centrifuged at 10,000× *g* at 4 °C for 5 min and resuspended in 1 mL of RO medium. Centrifugation was repeated twice. Thereafter, the pellet was transferred to 50 mL of RO medium and incubated over 7 h at 28 °C with continuous shaking. Samples were collected every 30 min and viral abundance was quantified using the double agar overlay plaque assay. The latent period was followed by a single burst of phages. The burst size was the average number of phages released per infected host cell and calculated as the ratio between the number of phages before and after the burst [35].

### 2.5. Lipid in the Viral Capsid

To investigate the presence of lipid in the viral capsid, 1 mL of phages were mixed by vibrating with 0 μL, 20 μL and 200 μL of chloroform, respectively, for 1 min and then kept at room temperature for 30 min. After centrifuging at a slow speed, phages remaining in the supernatant were dropped onto a *D. shibae* DFL12^T^ plate. The result was determined by the emergence of plaques.

### 2.6. DNA Extraction

Phage DNA was extracted using the phenol–chloroform extraction method. Briefly, purified phages were lysed by the addition of proteinase K (100 mg·mL^−1^), SDS (10%, wt/vol) and EDTA (0.5 mol·L^−1^, pH 8.0) and incubated at 55 °C in water for 3 h. The digested sample was then added to an equal volume of phenol/chloroform/isoamyl alcohol (25:24:1) and centrifuged at 12,000× *g* and 4 °C for 5 min to remove any debris. This step was repeated twice. The supernatant was sequentially purified by adding chloroform/isoamyl alcohol (24:1) and centrifuging at 12,000× *g* and 4 °C for 10 min. Following this, the supernatant was mixed with isoamyl alcohol and kept at −20 °C overnight. The precipitate was allowed to air-dry after slowly flushing with cold 70% ethanol. Samples were resuspended in 100 μL TE buffer (10 mM Tris-HCl, 1 mM EDTA, pH 8.0) and stored at 4 °C before analysis.

### 2.7. Genome Sequencing and Analysis, and Phylogenetic Analyses

The genomic DNA of R5C was sequenced on the Illumina Hiseq 2500 platform using the TruSeq PE Cluster Kit (Illumina, San Diego, CA, USA). The sequences were assembled using Velvet software (v1.2.03) (4699× coverage) [36]. A termini analysis was used to identify the phage’s termini and genome packaging [37]. The raw reads were aligned to the genome sequence using a CLC Genomics Workbench (version 3.6.1). The GeneMarkS online server and ORF Finder were used to predict open reading frames (ORFs), while tRNAscan-SE was used to identify tRNA sequences [38,39,40]. Translated ORFs were analyzed and annotated by the algorithms of a BLASTP search against the NCBI database with E-value ≤ 10^−3^ [41,42]. Gene maps were created based on the genome annotations using the Java Operon. DNA polymerase I was used to investigate the phage R5C phylogeny. A maximum-likelihood method in the MEGA 6.0 software package was used to construct the phylogenetic tree.

The complete genome sequence was submitted to the GenBank database under the accession number KY606587.

### 2.8. Recruitment of Metagenomic Data

For a better understanding of the geographical distribution of R5C, the phage genes were used as queries to search against metagenomic databases of the Pacific Ocean Virome (POV) and Global Ocean Survey (GOS) [43,44]. Samples in POV were collected from various seasons (spring, summer, fall, and winter), depths (10 m to 4300 m), and proximities to land (coastal to open ocean). Samples in GOS were taken from a wide variety of aquatic habitats collected over 8000 km. Only sequences with a threshold E-value of 10^−^^5^ and a minimum amino acid length of 30 and a bit score greater than 40 were extracted from the database. In addition, we searched for homologs of the R5C genome in the Integrated Microbial Genomes/Virus (IMG/VR) database and the Environmental Viral Genomes databases (EVGs) generated by Nishimura et al. (2017) [45,46]. To test any spacers of CRISPR array within the phage sequence, the genome of phage was searched against viral spacer database of IMG/VR, as well as in CRISPRs loci of bacterial isolates used in the present study [47].

## 3. Results and Discussion

### 3.1. Isolation and Characterization of Phage R5C

**Figure 1:**
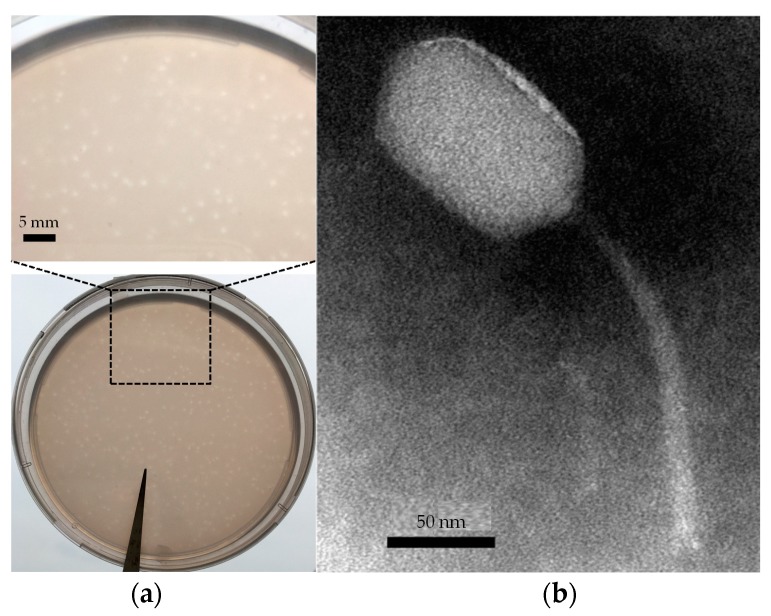
(**a**) Plaques and (**b**) transmission electron microscopy image of phage R5C.

Phages infecting *D. shibae* DFL12^T^ were isolated from seawater from the oligotrophic South China Sea and designated as vB_DshS-R5C based on nomenclature suggested by Kropinski et al. (2009) [48]. Plaques resulting from R5C lysis appeared small and blurry, with a diameter of 1–2 mm after 1–2 days incubation (Figure 1a). TEM showed that the phage R5C had a long hexagon head that measured about 114 ± 2 nm in length and 70 ± 2 nm for the greatest width in addition to a flexional long tail with a length of 142 ± 2 nm (Figure 1b). Based on its morphology, R5C belongs to the *Siphoviridae* family from the order *Caudovirales*.

Host infectivity was analyzed for 19 bacterium strains. The host infectivity test demonstrated that the phage R5C possesses a narrow host range and is only able to infect *D. shibae* DFL12^T^ (Table 1). Generally, myoviruses display the broadest host range among the three families of tailed dsDNA viruses, while podoviruses display the narrowest [1]. Interestingly, a narrow host range was observed for siphoviruses DSS3Ф8 and RDJLФ1, while a broad host range was observed for podoviruses DS-1410Ws-06, RD-1410W1-01 and RD-1410Ws-07 (Table 1). This confirms the specificity and complexity of phage–host interactions and the rich diversity of roseophages. The phage R5C was resistant to all three concentrations of chloroform. These results indicated the absence of lipids in the capsid or the surrounding lipid layer, which has commonly been observed among all of the roseophages isolated so far.

### 3.2. Life Cycle

**Figure 2:**
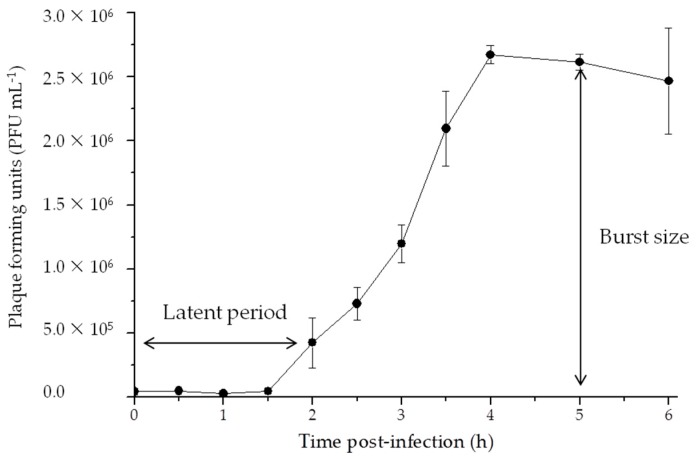
One-step growth curve of the phage R5C.

The results from the one-step growth curve are shown in Figure 2. The latent period for the phage R5C lasted about 1.5–2 h and a growth plateau was reached in 4 h (Table S1). R5C exhibited a small burst size of 65 PFU cell^−1^. The burst size of roseophages appeared to be highly variable, ranging from 10 cell^−1^ to 1500 cell^−1^. It should be noted that different methods were used for the enumeration of viral abundance. Generally, burst size is thought to be influenced by a number of factors such as bacterial/viral size, the metabolic activity of the host as well as the characteristic of the phage and host. A correlation between burst size and the trophic status of the environment has also been proposed [49,50]. However, this needs to be further verified because some phages (e.g., RPP1 and RD-1410Ws-01), which are isolated from eutrophic environments, also exhibit low burst sizes. Furthermore, even though R5C and DS-1410Ws-06 were isolated from the same host and cultured in a similar nutrient-rich medium, the burst sizes were different [32]. Similar to the host’s physiological and ecological characteristics, features affecting phage burst size should not be ignored.

### 3.3. Genome Features

**Figure 3:**
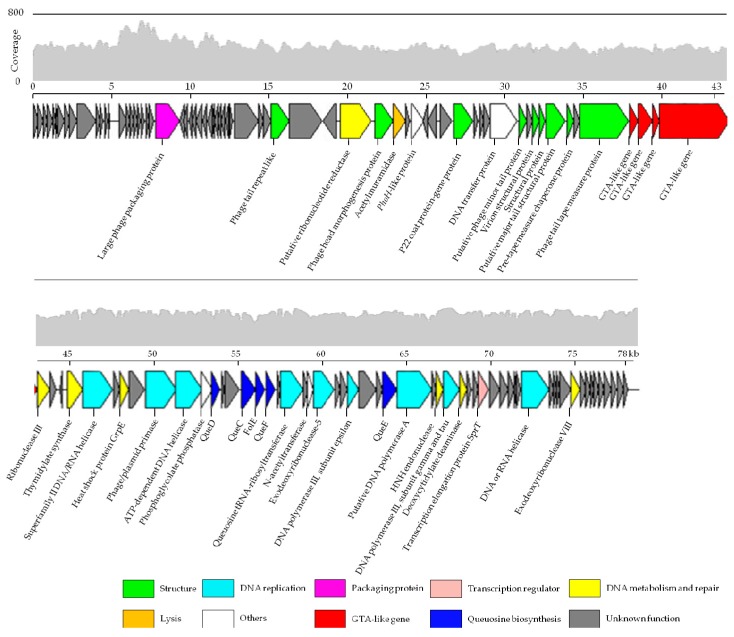
Genome map of the roseophage R5C. ORFs are depicted by leftward or rightward oriented arrows according to the direction of transcription. Gene features and genome modules (structure, lysis and DNA replication) are color-coded according to the legend below the figure.

The terminal analysis revealed that no protruding cohesive end was found in the complete genome and suggested that phage R5C has a circular, double-stranded DNA genome according to Zhang et al. [37]. The genome size of R5C is 77,874 bp (Figure 3), which is the second largest among the published genomes of roseophages. It is suggested that the likelihood of phage interference with host cellular activities increases with genome size. R5C has a G+C content of 61.5%, which is the highest among all roseophages (Table S1). Generally, the G+C content is lower in phages than that in their hosts, while temperate phages have smaller biases towards G+C content [51]. For example, the average G+C values of the temperate phages ΦCB2047-A (58.8%) and ΦCB2047-C (59.0%) are close to that of their host *Sulfitobacter* sp. strain 2047 (60.3%). Interestingly, a small G+C deviation is also observed between R5C and its host (66.0%), which suggests that R5C may follow a temperate phage strategy. No tRNA sequences were detected in the R5C genome using the tRNAscan-SE program. The lack of tRNA was also found in other roseophages such as SIO1, P12053L, ΦCB2047-A, ΦCB2047-C, RDJLΦ1, RDJLΦ2, RD-1410W1-01, RD-1410Ws-07 and DS-1410Ws-06. Among the four roseosiphophages, DSS3Φ8 has the longest genome containing 24 tRNAs. In the literature, tRNA has been associated with longer genome length, higher codon usage bias and higher virulence [52].

In total, 123 ORFs were identified in the R5C genome using GeneMarkS and ORF Finder software (Table S2). A total of 66 gene products had homologous sequences in the NCBI non-redundant protein database and 41 of these could be assigned a recognizable function. At the amino acid level, genes homologous to that from other phages showed less than 71% similarity. About 66.7% of the ORFs (82 ORFs, about 40% of the phage genome length) had no annotated features, while 57 of these ORFs had no matches in the databases. Single gene analysis showed R5C to be weakly similar to the known *Siphoviridae*. However, little or no nucleotide similarity was detected with these phages and protein homology was also detected with a few loci, with only one or two signature phage genes being shared between phages. Fifteen ORFs of R5C were homologous to that of both RDJLФ1 and RDJLФ2, showing similarly low identity levels (ranging from 24 to 72% and 25 to 74%, respectively). Furthermore, 19 ORFs with low identity (22–51%) were detected to be similar between R5C and DSS3Ф8. This suggested that R5C sequences presented high levels of divergence from known phage genomes and that proteins encoded by siphoviruses are under-represented in the database.

Among the 41 ORFs with recognizable functions, 10 were related to the structure and assembly of virions, such as a coat protein, a head-to-tail connecting protein, a tail fiber protein and the large subunit of terminase. Sixteen ORFs were predicted to encode proteins involved in DNA replication, metabolism and repair, while one conserved lysis ORF, acetylmuramidase, was predicted in the R5C genome. This was the first time that the DNA transfer protein, which is transcribed in the pre-early stage of infection in T5, had been detected in roseophages. Interestingly, four gene transfer agent (GTA) homologous genes and five queuosine biosynthesis genes were found in the R5C genome. Additionally, integrase and repressor genes, which indicate a potential for a lysogenic cycle, were not found in the R5C genome.

**Figure 4:**
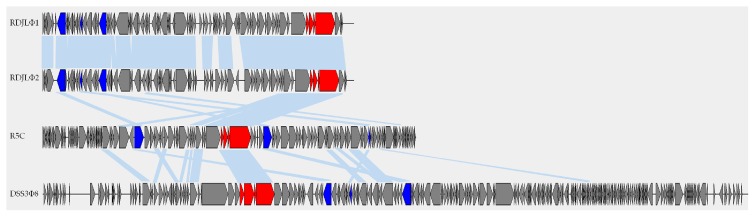
Comparison of four roseophages that possess GTA genes. Four GTA genes are indicated in red and other three conserved shared genes are indicated in blue. Genes sharing amino acid similarity between two phages are connected by light blue lines (E-value < 10^−^^3^).

We compared the genomes of four roseosiphophages that possess gene transfer agent genes and found only seven conserved shared genes, including ribonucleotide reductase, DNA helicase, deoxycytidylate deaminase and GTA-like genes, with 22–50% identity at the amino acid level (Figure 4). This demonstrated the extremely high level of genetic divergence of roseosiphophages. The ribonucleotide reductase gene in R5C shares high amino acid identity with that of roseophages RDJLФ1 (44%) and RDJLФ2 (44%). As a key enzyme involved in DNA synthesis, ribonucleotide reductases are found in all organisms and convert nucleotides into deoxynucleotides [53]. In the phosphorus-limited marine environment, obtaining sufficient free nucleotides is critical for DNA synthesis [54,55]. DNA helicases are motor proteins that use the energy from NTP hydrolysis to separate transiently energetically-stable duplex DNA into single strands [56]. The ubiquity of helicases in prokaryotes, eukaryotes, and viruses indicates their fundamental importance in DNA metabolism [57]. Deoxycytidylate deaminases catalyze the deamination of dCMP to dUMP and thus provide the nucleotide substrates for thymidylate synthase [58]. All roseosiphophages isolated have highly conserved GTA-like genes, whereas all podophages infecting the *Roseobacter* clade roseopodophages lack similar genes. The four GTA-like genes (gp12–gp15) are close to genes encoding structural proteins, such as the tail tape measure protein of R5C, and the same structural phenomenon is also observed in other GTA-harboring phage genomes. These observations suggested that the function of gp12–gp15 may be related to the specific structure of siphophages, such as the tail. Further protein analyses are needed to verify this assumption.

Like many other phages, the R5C genome contains a variety of auxiliary metabolic genes (AMGs). Currently, DNA metabolism and nucleotide synthesis genes are the most prevalent AMGs in roseophage. In R5C, we found AMGs frequently appeared in marine phages, such as *phoH* (ORF 47) and those firstly identified in roseophages (e.g., heat shock protein (ORF 74) and queuosine biosynthesis genes (ORF 79, ORF 82–84 and ORF 95)). A greater number of AMGs may broaden the role that phage play in their hosts’ fitness during infection.

The *phoH* gene has been detected in phages infecting both heterotrophic and autotrophic bacteria, such as *Prochlorococcus* phage P-SSM2, *Synechococcus* phage Syn9, SAR11 phage HTVC008M, and *Vibrio* phage KVP40 [6,54,59,60]. Roseophage SIO1 and DSS3Ф8 also possess the *phoH* gene [13,26]. Phage-encoded *phoH* genes have previously been described as apparent parts of a multi-gene family with divergent functions and have played a part in phospholipid metabolism, RNA modification, and fatty acid beta-oxidation [54,61,62]. It is suggested that the *phoH* gene in the phages aids host regulation of phosphate uptake and metabolism under low-phosphate conditions, which is consistent with the environment from which R5C was isolated, namely the oligotrophic South China Sea.

Heat shock proteins are postulated to protect organisms from the toxic effects of heat and other forms of stress. These proteins exist in every organism studied from archaebacteria to eubacteria and from plants to animals [63]. Cellular heat-shock responses occur during the replication of many viruses, such as adenovirus and human cytomegalovirus [64,65]. This is the first report of a heat shock protein in roseophages. The *grpE* gene alone encodes a 24-kDa heat shock protein. The GrpE heat shock protein is important for bacteriophage λ DNA replication at all temperatures and for bacterial survival under certain conditions [66].

As a hypermodified nucleoside derivative of guanosine, queuosine occupies the wobble position (position 34) of the tRNAs coding for Asp, Asn, His or Tyr. The hypomodification of queuosine-modified tRNA plays an important role in cellular proliferation and metabolism [67]. The mechanisms of action of the queuosine biosynthesis genes in viruses remain unclear, even though similar gene clusters have been found in *Streptococcus* phage Dp-1, *Escherichia coli* phage 9g and other viruses [68,69,70]. The queuosine biosynthesis genes were detected for the first time in the genome of a roseophage in this study.

### 3.4. Phylogenetic Analyses

**Figure 5:**
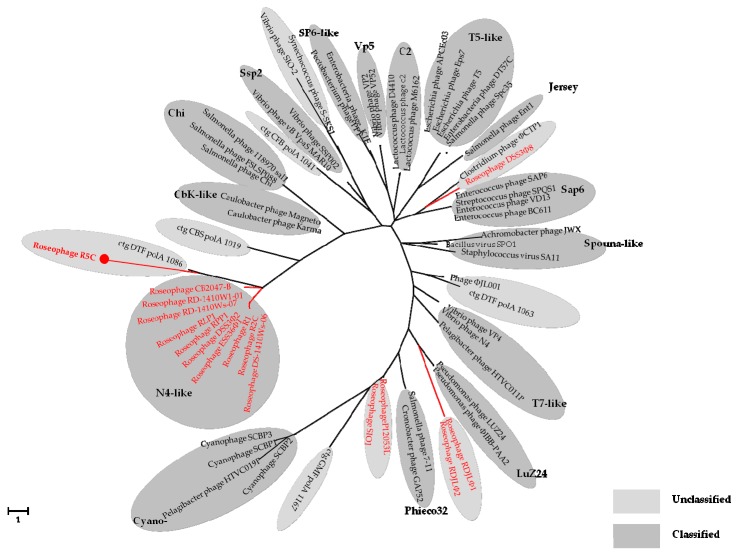
Unrooted maximum likelihood phylogenetic tree of DNA polymerase I of bacteriophages. Red color represents roseophages.

DNA polymerases, which play essential roles in viral replication, are found in many tailed bacteriophages, with three conserved motifs (motifs A, B, C) being present in all virioplankton metagenomic DNA polymerases [71]. In motif B, a link has been reported between leucine or tyrosine substitution in the site corresponding to phenylalanine and the phage lifestyle [72]. For example, all of the cultured phages with the tyrosine substitution were lytic, whereas lysogenic phages carrying the *pol*A gene possessed the leucine substitution. However, no evidence was found to link DNA polymerase I with the biological requirements for a lysogenic or lytic life cycle in R5C. Phylogenetic analyses based on DNA polymerase I showed that R5C was distantly related to other roseophages, most of which fell into the “N4-like” cluster. On the DNA polymerase I phylogenetic tree, R5C was most closely related to ctg DTF polA 1086, which was an environmental DNA polymerase sequence from Dry Tortugas surface water (Figure 5). Based on the currently available data, it is difficult to determine the taxonomic classification of R5C.

### 3.5. Environmental Distribution

**Figure 6:**
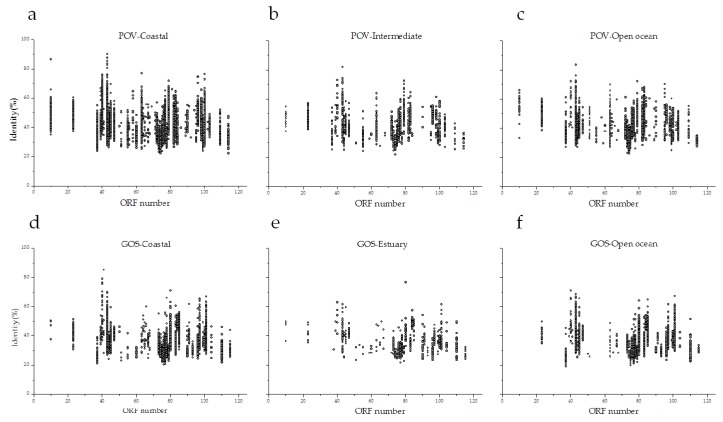
Prevalence of R5C-like ORFs in environmental viral metagenomic data. (**a**) Coastal metagenomes of POV; (**b**) Intermediate metagenomes of POV; (**c**) Open ocean metagenomes of POV; (**d**) Coastal metagenomes of GOS; (**e**) Estuary metagenomes of GOS; (**f**) Open ocean metagenomes of GOS.

To assess whether phage R5C is common in the environments, the R5C genome was searched against the IMG/VR and EVGs databases, which are assembled from ecologically diverse metagenomic samples [45,46]. However, no contig and genome with similarity to phage R5C was detected from the IMG/VR and EVGs databases. In addition, the genome of R5C was searched against spacers within CRISPRs of its host DFL12^T^, the isolates used in this study, and the viral spacer database of IMG/VR. None of these analyses showed any match between R5C and viral spacer sequences within CRISPRs. When the R5C genes were searched against the POV and GOS databases, we found that their homologs were widespread from coastal regions to open oceans, similar to the previously shown genomic recruitment of N4-like roseophages [29]. A more detailed analysis showed that similar levels of identity (coastal, 22.2–90.0%; intermediate, 22.0–81.8%; open ocean, 22.6–83.3%) were found in different kinds of environment in the POV. Surprisingly, the highest frequency of counts was observed in samples from coastal areas in both the POV and GOS databases, despite the fact that R5C was isolated from open water (Figure 6). This distribution pattern was consistent with that found in previously published studies for roseophages DSS3Ф2 and EE36Ф1, but not RPP1 [16,29]. RPP1 was isolated from a coastal area but showed a higher distribution in open oceans [29]. These contrasting distribution patterns to isolation environments indicated that the interaction between roseophages and their hosts may be more complex and dynamic than previously thought, with further studies needed to reveal the global pattern of roseophages and their ecological significance.

## 4. Conclusions

Taken together, physiological and genomic characterization suggested that the phage R5C is a novel lytic *Siphoviridae* roseophage. Our study demonstrated that oligotrophic open ocean is also a source of roseophages, with novel roseophages possibly existing in this environment. The data for R5C provide valuable insight into our understanding of roseosiphophages, which have so far been under-investigated. However, these findings have raised several questions worth exploring in future studies: (1) What is the distribution pattern of roseophages and their major groups? (2) Do environmental conditions, such as nutrient levels, impact the ecological behavior of roseophages? and (3) What is the evolutionary forcing driving diversity of roseophage? With an increasing number of roseophages being isolated and considering the ecological significance of *Roseobacter*, *Roseobacter*–phages may serve as a model system for studying the interaction between marine bacteria and viruses. Tackling the questions above will expand our knowledge in this field.

## Figures and Tables

**Table 1:** Host range of roseophages (+: infected; −: uninfected).

Strains	R5C	DSS3Φ8	RDJLФ1	RLP1	RPP1	DSS3Φ2	ESS36Φ1	DS-1410Ws-06	RD-1410W1-01	RD-1410Ws-07
*D. shibae* DFL12^T^	+							+	+	+
*Acinetobacter* sp. JL1404			−							
*Alcanivorax* sp. JL1378			−							
*Alteromonas* sp. JL1357			−							
*Antarctobacter* sp. JL351			−							
*Citromicrobium* sp. JL1363	−									
*Citromicrobium* sp. JL1351	−		−							
*Citromicrobium* sp. JL2201	−									
*Citromicrobium* sp. JL354	−		−							
*Citromicrobium* sp. WPS32	−									
*Cytophaga* sp. JL1362			−							
*Dinoroseobacter* sp. JL1447	−		−							
*Erythrobacter litoralis* DMS 8509	−		−							
*Erythrobacter longus* DSM 6997	−		−							
*Erythrobacter* sp. JL1350			−							
*Erythrobacter* sp. JL359			−							
*Erythrobacter* sp. JL475.	−		−							
*Furvibacter* sp. JL1383			−							
*Hoeflea phototrophica* DFL-43^T^	−									
*Leisingera methylohalidivorans* MB2				−	−					
*Marinovum algicola* ATCC 51440^T^				−	−					
*Micrococcus* sp. JL1389			−							
*Nocardioides* sp. JL1369			−							
*Paenibacillus* sp. JL1210	−									
*Phaeobacter* sp. 27–4						−	−			
*Pseudoalteromonas* sp. JL1391			−							
*Rhodobacteraceae* sp. 176				−	−					
*Roseobacter denitrificans* ATCC 33942						−	−			
*Roseobacter denitrificans* OCh 114	−	−	+					+	+	+
*Roseobacter litoralis* ATCC 49566						−	−			
*Roseobacter litoralis* DMS 6996			−							
*Roseobacter litoralis* Och149								−	+	+
*Roseobacter* sp. JL1336			−							
*Roseobacter* sp. TM1038						−	−			
*Roseobacter* sp. TM1039						−	−			
*Roseomonas* sp. JL2290	−									
*Roseomonas* sp. JL2293	−									
*Roseovarius* sp.TM1042						−	−			
*Roseovarius nubinhibens* ISM^T^		−		−	+	−	−	−	−	−
*Roseovarius* sp. 217				+	−					
*Roseovarius* sp. JL2434	−									
*Roseovarius crassostreae* CV919–312^T^				−	−					
*Roseovarius mucosus* DFL–24^T^				−	−					
*Ruegeria atlantica* AMA–03				−	−					
*Ruegeria* sp. 198				−	−					
*Ruegeria* sp. JL126	−									
*Sagittula stellata* E–37				−	−					
*Silicibacter pomeroyi* DSS3	−	+		−	−	+		+	+	+
*Silicibacter* sp. TM1040		−				−	−			
*Sphingobium* sp. JL1088	−									
*Stappia* sp. JL1358			−							
*Sulfitobacter* sp. 1921						−	−			
*Sulfitobacter pseudonitzschiae* H3								−	−	−
*Sulfitobacter* sp. EE–36		−	−				+	−	−	−
*Sulfitobacter* sp. JL1353			−							
*Sulfitobacter* sp CBB406						−	−			
*Sulfitobacter* sp. CHSB4						−	−			
*Sulfitobacter* sp. JL351						−	−			
*Sulfitobacter* sp. T11						−	−			

## Supplementary Materials

The following are available online at www.mdpi.com/1999-4915/9/5/109/s1, Table S1: Roseophages for which genome sequences are published; Table S2: Genome annotations of phage vB_DshS-R5C.
