# Erratum: Yang, Y.; et al. A Novel Roseosiphophage Isolated from the Oligotrophic South China Sea. *Viruses* 2017, *9*, 109

**DOI:** 10.3390/v11080734

**Published:** 2019-08-08

**Authors:** Yunlan Yang, Lanlan Cai, Ruijie Ma, Yongle Xu, Yigang Tong, Yong Huang, Nianzhi Jiao, Rui Zhang

**Affiliations:** 1 State Key Laboratory of Marine Environmental Science, Institute of Marine Microbes and Ecospheres, Xiamen University (Xiang’an), Xiamen 361102, China; 2 Beijing Institute of Microbiology and Epidemiology; State Key Laboratory of Pathogen and Biosecurity, Beijing 100071, China

The authors wish to make the following changes to their paper [1].

(1) Figure 5 should be replaced with:

(2) In Section 3.4. “Phylogenetic Analyses” within Results and Discussion, the sentence of “On the DNA polymerase I phylogenetic tree, R5C was most closely related to ctg DTF polA 1086, which was an environmental DNA polymerase sequence from Dry Tortugas surface water (Figure 5)” should be corrected as “On the DNA polymerase I phylogenetic tree, R5C formed a relatively single branch (Figure 5)”.

The changes were caused by a mistake during the sequence alignment.

The authors apologize for any inconvenience this may have caused. The manuscript will be updated and the original will remain online on the article webpage, with a reference to this erratum.

## Figures and Tables

**Figure 5:**
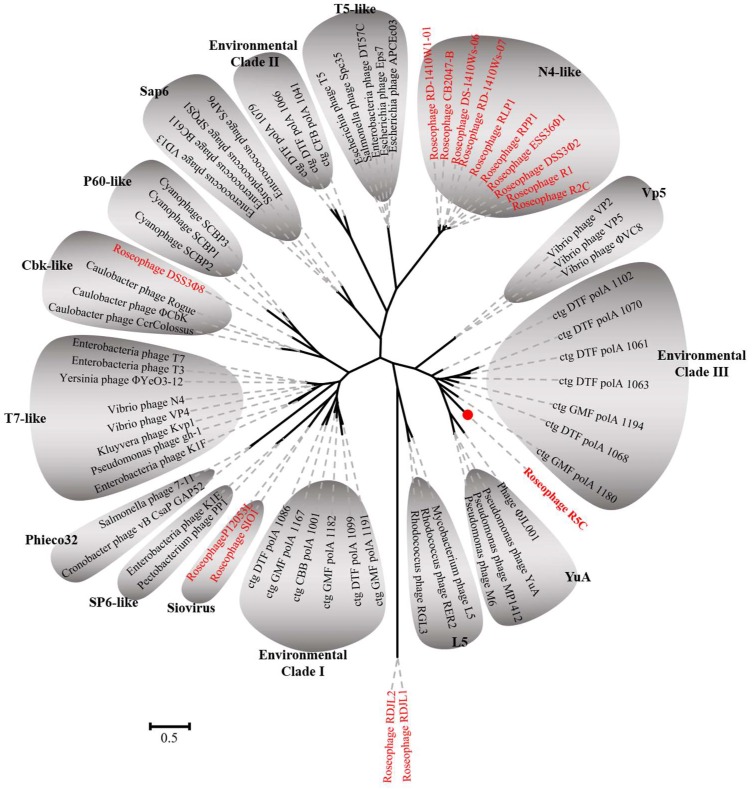
Unrooted maximum likelihood phylogenetic tree of DNA polymerase I of bacteriophages. Red color represents the roseophages. The sequences were aligned by ClustalW and Bootstrap = 1000.
