# Supplementary material for: A Novel Roseosiphophage Isolated from the Oligotrophic South China Sea
Source: Viruses. 2017 May 15;9(5):109. doi: 10.3390/v9050109 (PMC5454422; doi:10.3390/v9050109)
Supplement: Supplementary file 1 [file viruses-09-00109-s001.doc]

Supplementary Materials: A Novel Roseosiphophage Isolated from the Oligotrophic South China Sea

**Yunlan Yang, Lanlan Cai, Ruijie Ma, Yongle Xu, Yigang Tong, Yong Huang, Nianzhi Jiao and Rui Zhang**

**Table S1.** Roseophages for which genome sequences are published.

| **Phage** | **Host range** | **Isolation conditions** | **Family** | **Latent period (h)** | **Burst size (cell^-1^)** | **Genome size (kb)** | **G+C (%)** | **No. of tRNAs** | **GTA-like genes** | **References** |
| --- | --- | --- | --- | --- | --- | --- | --- | --- | --- | --- |
| SIO1 | *Roseobacter* SIO67 | Eutrophic | *Podoviridae* |  |  | 39.9 | 46.2 | 0 |  | Rohwer *et al.* (2000) |
| P12053L | *Celeribacter* sp. strain IMCC12053 | Eutrophic | *Caudovirales* |  |  | 35.9 | 46.1 | 0 |  | Kang *et al*. (2012) |
| ΦCB2047-B | *Sulfitobacter* sp. strain 2047 | Eutrophic | *Podoviridae* |  |  | 74.5 | 43 | 15 |  | Ankrah *et al*. (2014a) |
| ΦCB2047-A | *Sulfitobacter* sp. strain 2047 | Eutrophic | *Podoviridae* |  |  | 40.9 | 58.8 | 0 |  | Ankrah *et al.* (2014b) |
| ΦCB2047-C | *Sulfitobacter* sp. strain 2047 | Eutrophic | *Podoviridae* |  |  | 40.9 | 59 | 0 |  | Ankrah *et al.* (2014c) |
| RLP1 | *Roseovarius* sp. 217 | Eutrophic | *Podoviridae* | 4-6 | 100 | 74.6 | 49 | 3 |  | Chan *et al*. (2014) |
| RPP1 | *Roseovarius nubinhibens* | Eutrophic | *Podoviridae* | 4-6 | 10 | 74.7 | 49.1 | 3 |  | Chan *et al*. (2014) |
| RDJLΦ1 | *Roseobacter denitrificans* OCh114 | Oligotrophic | *Siphoviridae* | 1-1.5 | 203 | 62.7 | 57.9 | 0 | GTA gp12, 13,14,15 | Huang *et al.* (2011) |
| RDJLΦ2 | *Roseobacter denitrificans* OCh114 | Eutrophic | *Siphoviridae* |  |  | 63.5 | 57.3 | 0 | GTA gp12, 13,14,15 | Liang *et al*. (2016) |
| ESS36Φ1 | *Sulfitobacter* sp. EE36 | Eutrophic | *Podoviridae* | 2 | 1500 | 73.3 | 47 | 3 |  | Zhao *et al.* (2009) |
| DSS3Φ2 | *Ruegeria pomeroyi* DSS3 | Eutrophic | *Podoviridae* | 3 | 350 | 74.6 | 47.9 | 3 |  | Zhao *et al*. (2009) |
| DSS3Φ8 | *Ruegeria pomeroyi* DSS3 | Eutrophic | *Siphoviridae* | 2 | 120 | 146.1 | 56 | 24 | GTA gp12, 13,14,15 | Zhan *et al.* (2016) |
| RD-1410W1-01 | *Roseobacter denitrificans* OCh114; *Dinoroseobacter shibae* DFL12^T^; *Roseobacter litoralis OCh149; Ruegeria pomeroyi* DSS3 | Eutrophic | *Podoviridae* | 1-2 | 27 | 72.7 | 49.5 | 0 |  | Li *et al*. (2016) |
| RD-1410Ws-07 | *Roseobacter denitrificans* OCh114; *Dinoroseobacter shibae* DFL12^T^; *Roseobacter litoralis OCh149; Ruegeria pomeroyi* DSS3 | Eutrophic | *Podoviridae* | 1-2 | 341 | 76.3 | 50 | 0 |  | Li *et al*. (2016) |
| DS-1410Ws-06 | *Dinoroseobacter shibae* DFL12^T^; *Roseobacter denitrificans* OCh114; *Ruegeria pomeroyi* DSS3 | Eutrophic | *Podoviridae* | 1-2 | 298 | 76.3 | 50 | 0 |  | Li *et al*. (2016) |
| R1 | *Dinoroseobacter shibae* DFL12^T^ | Eutrophic | *Podoviridae* |  |  | 75.0 | 49.3 | 2 |  | Ji *et al.* (2015) |
| R2C | *Dinoroseobacter shibae* DFL12^T^; *Roseobacter denitrificans* OCh114 | Eutrophic | *Podoviridae* |  |  | 74.8 | 49.2 | 2 |  | Cai *et al*. (2015) |
| R5C | *Dinoroseobacter shibae* DFL12^T^ | Oligotrophic | *Siphoviridae* | 1.5-2 | 65 | 77.9 | 61.5 | 0 | GTA gp12, 13,14,15 | This study |

GTA: gene transfer agent.

**Table** **S2.** Genome annotations of phage vB_DshS-R5C.

| **ORF no.** | **Strand** | **Start** | **End** | **Best Homologs** | **Accession** | [**E value (<10^-3^)**](http://blast.ncbi.nlm.nih.gov/Blast.cgi?CMD=Get&ALIGNMENTS=100&ALIGNMENT_VIEW=Pairwise&CDD_SEARCH_STATE=1&DATABASE_SORT=0&DESCRIPTIONS=100&DYNAMIC_FORMAT=on&FIRST_QUERY_NUM=0&FORMAT_OBJECT=Alignment&FORMAT_PAGE_TARGET=&FORMAT_TYPE=HTML&GET_SEQUENCE=yes&I_THRESH=&LINE_LENGTH=60&MASK_CHAR=2&MASK_COLOR=1&NEW_VIEW=yes&NUM_OVERVIEW=100&OLD_BLAST=false&PAGE=Proteins&QUERY_INDEX=0&QUERY_NUMBER=0&RESULTS_PAGE_TARGET=&RID=GV5C9JFX01R&SHOW_LINKOUT=yes&SHOW_OVERVIEW=yes&STEP_NUMBER=&WORD_SIZE=3&OLD_VIEW=false&DISPLAY_SORT=0&HSP_SORT=0) | [**aa identity**](http://blast.ncbi.nlm.nih.gov/Blast.cgi?CMD=Get&ALIGNMENTS=100&ALIGNMENT_VIEW=Pairwise&CDD_SEARCH_STATE=1&DATABASE_SORT=0&DESCRIPTIONS=100&DYNAMIC_FORMAT=on&FIRST_QUERY_NUM=0&FORMAT_OBJECT=Alignment&FORMAT_PAGE_TARGET=&FORMAT_TYPE=HTML&GET_SEQUENCE=yes&I_THRESH=&LINE_LENGTH=60&MASK_CHAR=2&MASK_COLOR=1&NEW_VIEW=yes&NUM_OVERVIEW=100&OLD_BLAST=false&PAGE=Proteins&QUERY_INDEX=0&QUERY_NUMBER=0&RESULTS_PAGE_TARGET=&RID=GV5C9JFX01R&SHOW_LINKOUT=yes&SHOW_OVERVIEW=yes&STEP_NUMBER=&WORD_SIZE=3&DISPLAY_SORT=3&HSP_SORT=3) | **Best Domain Hits (E-value<10^-3^)/Accession** | **Putative Function** |
| --- | --- | --- | --- | --- | --- | --- | --- | --- | --- |
| 1 | + | 5 | 301 | None | n/a | n/a | n/a |  | Hypothetical protein |
| 2 | + | 292 | 618 | None | n/a | n/a | n/a |  | Hypothetical protein |
| 3 | + | 615 | 863 | None | n/a | n/a | n/a |  | Hypothetical protein |
| 4 | + | 860 | 1249 | None | n/a | n/a | n/a |  | Hypothetical protein |
| 5 | + | 1251 | 1424 | None | n/a | n/a | n/a |  | Hypothetical protein |
| 6 | + | 1421 | 1549 | Roseovarius sp. 217 phage 1 | [CBW47003.1](https://www.ncbi.nlm.nih.gov/protein/308516898?report=genbank&log$=prottop&blast_rank=1&RID=DMJYXGXV016) | 4.00E-12 | 71% |  | Hypothetical protein |
| 7 | + | 1546 | 1956 | Leisingera sp. ANG-M7 | [WP_039184058.1](https://www.ncbi.nlm.nih.gov/protein/746118275?report=genbank&log$=prottop&blast_rank=1&RID=DMK9ASVT013) | 7.00E-09 | 41% |  | Hypothetical protein |
| 8 | + | 1937 | 2281 | Mesorhizobium ciceri | [WP_027037897.1](https://www.ncbi.nlm.nih.gov/protein/652693842?report=genbank&log$=prottop&blast_rank=1&RID=DMKD15H0013) | 5.00E-22 | 46% |  | Hypothetical protein |
| 9 | + | 2274 | 2735 | Rhizobium phage vB_RleM_P10VF | [YP_009099929.1](https://www.ncbi.nlm.nih.gov/protein/712913097?report=genbank&log$=prottop&blast_rank=1&RID=DMKKFX4D013) | 3.00E-14 | 45% |  | Hypothetical protein |
| 10 | + | 2748 | 3944 | Oceanicola sp. HL-35 | [WP_051469457.1](https://www.ncbi.nlm.nih.gov/protein/916862401?report=genbank&log$=prottop&blast_rank=1&RID=DMMX92R2016) | 8.00E-51 | 65% |  | Hypothetical protein |
| 11 | + | 3941 | 4171 | Salinispora arenicola | [WP_029022494.1](https://www.ncbi.nlm.nih.gov/protein/655980249?report=genbank&log$=prottop&blast_rank=1&RID=DMNRP5RP013) | 4.00E-08 | 36% |  | Hypothetical protein |
| 12 | + | 4173 | 4358 | None | n/a | n/a | n/a |  | Hypothetical protein |
| 13 | + | 4443 | 4676 | None | n/a | n/a | n/a |  | Hypothetical protein |
| 14 | + | 4687 | 4800 | None | n/a | n/a | n/a |  | Hypothetical protein |
| 15 | + | 5380 | 5823 | None | n/a | n/a | n/a |  | Hypothetical protein |
| 16 | + | 5816 | 6055 | None | n/a | n/a | n/a |  | Hypothetical protein |
| 17 | + | 6048 | 6293 | None | n/a | n/a | n/a |  | Hypothetical protein |
| 18 | + | 6296 | 6532 | Oceanicola sp. MCTG156(1a) | [WP_036556306.1](https://www.ncbi.nlm.nih.gov/protein/738647448?report=genbank&log$=prottop&blast_rank=1&RID=DMPBC3HS016) | 5.00E-06 | 48% |  | Hypothetical protein |
| 19 | + | 6529 | 6678 | None | n/a | n/a | n/a |  | Hypothetical protein |
| 20 | + | 6766 | 6915 | None | n/a | n/a | n/a |  | Hypothetical protein |
| 21 | + | 7025 | 7234 | None | n/a | n/a | n/a |  | Hypothetical protein |
| 22 | + | 7227 | 7544 | None | n/a | n/a | n/a |  | Hypothetical protein |
| 23 | + | 7631 | 9136 | Rhizobium sp. Root651 | [WP_062653803.1](https://www.ncbi.nlm.nih.gov/protein/1011834874?report=genbank&log$=prottop&blast_rank=1&RID=DMPDXV45013) | 1.00E-138 | 51% | COG5323/COG5323 | Large phage packaging protein |
| 24 | - | 9154 | 9438 | None | n/a | n/a | n/a |  | Hypothetical protein |
| 25 | - | 9419 | 9655 | None | n/a | n/a | n/a |  | Hypothetical protein |
| 26 | - | 9739 | 9930 | None | n/a | n/a | n/a |  | Hypothetical protein |
| 27 | - | 9933 | 10175 | Roseobacter phage RD-1410W1-01 | [ANJ20752.1](https://www.ncbi.nlm.nih.gov/protein/1035522393?report=genbank&log$=prottop&blast_rank=1&RID=DMPHT1YF013) | 5.00E-07 | 41% |  | Hypothetical protein |
| 28 | - | 10172 | 10549 | None | n/a | n/a | n/a |  | Hypothetical protein |
| 29 | - | 10561 | 10827 | None | n/a | n/a | n/a |  | Hypothetical protein |
| 30 | - | 10888 | 11166 | None | n/a | n/a | n/a |  | Hypothetical protein |
| 31 | - | 11196 | 11330 | None | n/a | n/a | n/a |  | Hypothetical protein |
| 32 | - | 11330 | 11530 | None | n/a | n/a | n/a |  | Hypothetical protein |
| 33 | - | 11530 | 11742 | None | n/a | n/a | n/a |  | Hypothetical protein |
| 34 | - | 11742 | 11957 | None | n/a | n/a | n/a |  | Hypothetical protein |
| 35 | - | 12031 | 12213 | None | n/a | n/a | n/a |  | Hypothetical protein |
| 36 | + | 12241 | 12429 | None | n/a | n/a | n/a |  | Hypothetical protein |
| 37 | + | 12550 | 14073 | Sulfitobacter sp. EE-36 | [WP_005852732.1](https://www.ncbi.nlm.nih.gov/protein/492460234?report=genbank&log$=prottop&blast_rank=1&RID=DMPRUFR5016) | 6.00E-157 | 51% | DUF4055/pfam13264 | Hypothetical protein |
| 38 | + | 14073 | 14393 | None | n/a | n/a | n/a |  | Hypothetical protein |
| 39 | + | 14399 | 14821 | Methylococcaceae bacterium Sn10-6 | [WP_045777711.1](https://www.ncbi.nlm.nih.gov/protein/788039013?report=genbank&log$=prottop&blast_rank=1&RID=DMPX2S6G013) | 4.00E-19 | 44% |  | Hypothetical protein |
| 40 | + | 14834 | 15994 | Shimia haliotis | [SFK78014.1](https://www.ncbi.nlm.nih.gov/protein/1097640782?report=genbank&log$=prottop&blast_rank=1&RID=DMR1A4XW013) | 1.00E-30 | 66% | PTR/pfam12789 | Phage tail repeat like |
| 41 | + | 16005 | 18041 | Rhodobacter phage RcRhea | [YP_009213486.1](https://www.ncbi.nlm.nih.gov/protein/971760181?report=genbank&log$=prottop&blast_rank=1&RID=DMR4DRWZ016) | 5.00E-21 | 52% |  | Hypothetical protein |
| 42 | - | 18085 | 18948 | None | n/a | n/a | n/a |  | Hypothetical protein |
| 43 | + | 19180 | 21111 | [Pseudomonas phage PAE1](https://blast.ncbi.nlm.nih.gov/Blast.cgi#alnHdr_971762606) | [YP_009215729.1](https://www.ncbi.nlm.nih.gov/protein/971762606?report=genbank&log$=prottop&blast_rank=1&RID=DMRAUBZE016) | 0 | 51% | NrdJ_Z/TIGR02504 | Putative ribonucleotide reductase |
| 44 | + | 21356 | 22462 | Methylobacterium sp. B1 | [WP_051044621.1](https://www.ncbi.nlm.nih.gov/protein/916309575?report=genbank&log$=prottop&blast_rank=1&RID=DMRDVNY8013) | 7.00E-81 | 41% | Phage_Mu_F/pfam04233 | Phage head morphogenesis protein |
| 45 | + | 22472 | 23278 | Ruegeria sp. TM1040 | [WP_011538937.1](https://www.ncbi.nlm.nih.gov/protein/499858203?report=genbank&log$=prottop&blast_rank=1&RID=DMRK9SWF013) | 8.00E-74 | 57% | ZliS/COG3926 | Acetylmuramidase |
| 46 | + | 23278 | 23511 | None | n/a | n/a | n/a |  | Hypothetical protein |
| 47 | + | 23615 | 24361 | Methylobacterium sp. UNC378MF | [SDA09612.1](https://www.ncbi.nlm.nih.gov/protein/1087225398?report=genbank&log$=prottop&blast_rank=1&RID=DMRRKMG9013) | 1.00E-55 | 46% | PhoH/pfam02562 | PhoH-like protein |
| 48 | - | 24358 | 24603 | None | n/a | n/a | n/a |  | Hypothetical protein |
| 49 | - | 24652 | 25164 | None | n/a | n/a | n/a |  | Hypothetical protein |
| 50 | + | 25387 | 26154 | Providencia alcalifaciens | [WP_006660882.1](https://www.ncbi.nlm.nih.gov/protein/493711248?report=genbank&log$=prottop&blast_rank=1&RID=DMRTF976013) | 8.00E-44 | 41% |  | Hypothetical protein |
| 51 | + | 26232 | 27461 | [Betaproteobacteria bacterium ADurb.Bin341](https://blast.ncbi.nlm.nih.gov/Blast.cgi#alnHdr_1167193009) | [OQA28157.1](https://www.ncbi.nlm.nih.gov/protein/1167193009?report=genbank&log$=prottop&blast_rank=5&RID=DMRX1GAH013) | 8.00E-45 | 40% | P22_CoatProtein/pfam11651 | P22 coat protein-gene protein |
| 52 | + | 27528 | 27779 | None | n/a | n/a | n/a |  | Hypothetical protein |
| 53 | + | 27854 | 28120 | Agrobacterium rhizogenes | [WP_065115637.1](https://www.ncbi.nlm.nih.gov/protein/1040492950?report=genbank&log$=prottop&blast_rank=1&RID=DMS3YJNU016) | 4.00E-09 | 32% |  | Hypothetical protein |
| 54 | + | 28113 | 28559 | Jannaschia helgolandensis | [SEL26926.1](https://www.ncbi.nlm.nih.gov/protein/1094404926?report=genbank&log$=prottop&blast_rank=1&RID=DMS66DR7013) | 7.00E-05 | 32% |  | Hypothetical protein |
| 55 | + | 28559 | 30208 | Escherichia phage APCEc03 | [AKO61464.1](https://www.ncbi.nlm.nih.gov/protein/857289809?report=genbank&log$=prottop&blast_rank=23&RID=DMSAK2H1013) | 2E-107 | 42% |  | DNA transfer protein |
| 56 | + | 30301 | 30792 | [Haemophilus] parasuis | [WP_021116799.1](https://www.ncbi.nlm.nih.gov/protein/544684984?report=genbank&log$=prottop&blast_rank=24&RID=DMSG006E016) | 2.00E-05 | 33% |  | Putative phage minor tail protein |
| 57 | + | 30789 | 31166 | Pseudomonas sp. CB1 | [WP_040263675.1](https://www.ncbi.nlm.nih.gov/protein/749664506?report=genbank&log$=prottop&blast_rank=1&RID=DMSS301W013) | 1.00E-05 | 27% |  | Hypothetical protein |
| 58 | + | 31159 | 31572 | Pseudomonas phage PaMx25 | [ALH23810.1](https://www.ncbi.nlm.nih.gov/protein/936761762?report=genbank&log$=prottop&blast_rank=9&RID=DMSU9JDC016) | 5.00E-23 | 42% |  | Virion structural protein |
| 59 | + | 31569 | 31976 | Pseudomonas phage AN14 | [ANO57383.1](https://www.ncbi.nlm.nih.gov/protein/1041568261?report=genbank&log$=prottop&blast_rank=4&RID=DMSWZ2D6013) | 7.00E-09 | 31% | DUF4128/pfam13554 | Structural protein |
| 60 | + | 31995 | 33191 | Pseudomonas phage PaMx74 | [YP_009199465.1](https://www.ncbi.nlm.nih.gov/protein/971745308?report=genbank&log$=prottop&blast_rank=7&RID=DMT0H6N2013) | 7.00E-45 | 34% |  | Putative major tail structural protein |
| 61 | + | 33339 | 33740 | Caulobacter phage CcrColossus | [YP_006988351.1](https://www.ncbi.nlm.nih.gov/protein/414088172?report=genbank&log$=prottop&blast_rank=2&RID=DMT5Y1CC013) | 1.00E-13 | 33% |  | Putative pre-tape measure chaperone protein |
| 62 | + | 33761 | 34111 | Thalassobaculum salexigens | [WP_028793472.1](https://www.ncbi.nlm.nih.gov/protein/655389578?report=genbank&log$=prottop&blast_rank=1&RID=DMT817C7013) | 9.00E-05 | 29% |  | Hypothetical protein |
| 63 | + | 34119 | 37175 | Nitrosomonas marina | [SEN43237.1](https://www.ncbi.nlm.nih.gov/protein/1094436915?report=genbank&log$=prottop&blast_rank=1&RID=DMTBCH5T016) | 2.00E-84 | 54% | tape_meas_TP901/TIGR01760 | Phage tail tape measure protein |
| 64 | + | 37247 | 37792 | Rhodobacteraceae bacterium PD-2 | [WP_023850192.1](https://www.ncbi.nlm.nih.gov/protein/564608947?report=genbank&log$=prottop&blast_rank=3&RID=DMTE6489013) | 7.00E-66 | 54% | DUF2460/pfam09343 | Glycoside hydrolase family 24 |
| 65 | + | 37789 | 38670 | Cohaesibacter marisflavi | [SFN60816.1](https://www.ncbi.nlm.nih.gov/protein/1098288199?report=genbank&log$=prottop&blast_rank=1&RID=DMTST5YN016) | 4.00E-89 | 47% | DUF2163/pfam09931 | Phage conserved hypothetical protein |
| 66 | + | 38667 | 39086 | Caulobacter vibrioides | [WP_004624757.1](https://www.ncbi.nlm.nih.gov/protein/490762495?report=genbank&log$=prottop&blast_rank=1&RID=DMTV4Z2K016) | 3.00E-45 | 55% | phage_NlpC_fam/TIGR02219 | Phage cell wall peptidase |
| 67 | + | 39091 | 43473 | Paracoccus aminovorans | [SFH84639.1](https://www.ncbi.nlm.nih.gov/protein/1097673681?report=genbank&log$=prottop&blast_rank=1&RID=DMTXJP0E013) | 0 | 40% | GTA_TIM/pfam13547 | Putative phage tail protein |
| 68 | + | 43460 | 44173 | Paracoccus sanguinis | [WP_036706984.1](https://www.ncbi.nlm.nih.gov/protein/738816551?report=genbank&log$=prottop&blast_rank=2&RID=DMU167DC016) | 1.00E-69 | 47% | DUF2793/pfam10983 | Ribonuclease III |
| 69 | + | 44183 | 44578 | Rhizobium tropici | [WP_041677398.1](https://www.ncbi.nlm.nih.gov/protein/754002337?report=genbank&log$=prottop&blast_rank=1&RID=DMU4268R013) | 2.00E-07 | 38% |  | Hypothetical protein |
| 70 | - | 44767 | 44907 | None | n/a | n/a | n/a |  | Hypothetical protein |
| 71 | + | 45234 | 46088 | Methylobacterium sp. CCH5-D2 | [WP_066922991.1](https://www.ncbi.nlm.nih.gov/protein/1055254151?report=genbank&log$=prottop&blast_rank=1&RID=DMU6NYMC016) | 2.00E-72 | 47% | thyX/PRK00847 | Thymidylate synthase (FAD) |
| 72 | + | 46081 | 47850 | Uncultured Mediterranean phage | [ANS05656.1](https://www.ncbi.nlm.nih.gov/protein/1043236956?report=genbank&log$=prottop&blast_rank=3&RID=DMU90U4Z016) | 3.00E-91 | 34% | HepA/COG0553 | Superfamily II DNA/RNA helicase |
| 73 | + | 47886 | 48245 | Uncultured Mediterranean phage uvMED | [BAR36385.1](https://www.ncbi.nlm.nih.gov/protein/787065687?report=genbank&log$=prottop&blast_rank=1&RID=DMUE8BTX016) | 7.00E-05 | 30% |  | Hypothetical protein |
| 74 | + | 48242 | 48787 | Candidate division Zixibacteria bacterium RBG_16_53_22 | [OGC94293.1](https://www.ncbi.nlm.nih.gov/protein/1082990371?report=genbank&log$=prottop&blast_rank=1&RID=DMUH3TPU013) | 5.00E-19 | 32% | PRK14147/PRK14147 | Heat shock protein GrpE |
| 75 | + | 48813 | 49700 | Candidatus Accumulibacter sp. SK-01 | [KFB66065.1](https://www.ncbi.nlm.nih.gov/protein/668670556?report=genbank&log$=prottop&blast_rank=1&RID=DMUNFYFH016) | 2.00E-39 | 34% |  | Hypothetical protein |
| 76 | + | 49763 | 51541 | Uncultured Mediterranean phage uvMED | [BAR34751.1](https://www.ncbi.nlm.nih.gov/protein/787063792?report=genbank&log$=prottop&blast_rank=2&RID=DMUX0N1G016) | 2.00E-42 | 26% | COG4951/COG4951 | Phage/plasmid primase |
| 77 | + | 51528 | 53042 | Candidatus Accumulibacter sp. SK-01 | [KFB66063.1](https://www.ncbi.nlm.nih.gov/protein/668670554?report=genbank&log$=prottop&blast_rank=3&RID=DMV3DGS1013) | 2.00E-77 | 32% | UvrD/COG0210 | ATP-dependent DNA helicase PcrA |
| 78 | + | 53039 | 53641 | Rhizobium sp. PDC82 | [SDJ25522.1](https://www.ncbi.nlm.nih.gov/protein/1086977281?report=genbank&log$=prottop&blast_rank=1&RID=DMVAHHPV016) | 1.00E-27 | 38% | HAD_5-3dNT/cd02587 | Phosphoglycolate phosphatase |
| 79 | + | 53638 | 54081 | Bradyrhizobium manausense | [WP_080138890.1](https://www.ncbi.nlm.nih.gov/protein/1167826744?report=genbank&log$=prottop&blast_rank=1&RID=DMVCSFZ7016) | 3.00E-49 | 56% | PTPS/pfam01242 | 6-pyruvoyl tetrahydrobiopterin synthase |
| 80 | + | 54192 | 54353 | None | n/a | n/a | n/a |  | Hypothetical protein |
| 81 | + | 54412 | 55209 | Pseudomonas phage PAE1 | [YP_009215732.1](https://www.ncbi.nlm.nih.gov/protein/971762609?report=genbank&log$=prottop&blast_rank=1&RID=DMVERHFH013) | 5.00E-09 | 30% | DUF3489/pfam11994 | Hypothetical protein |
| 82 | + | 55344 | 56192 | Brackiella oedipodis | [WP_051532207.1](https://www.ncbi.nlm.nih.gov/protein/916925495?report=genbank&log$=prottop&blast_rank=1&RID=DMVJKDHD013) | 2.00E-95 | 60% | QueC/pfam06508 | 7-cyano-7-deazaguanine synthase |
| 83 | + | 56192 | 56755 | Pseudomonas phage PaMx25 | [ALH23793.1](https://www.ncbi.nlm.nih.gov/protein/936761745?report=genbank&log$=prottop&blast_rank=1&RID=DMVK00JF013) | 5.00E-80 | 63% | folE/PRK09347 | Putative GTP cyclohydrolase |
| 84 | + | 56764 | 57348 | Caenispirillum salinarum | [WP_009539263.1](https://www.ncbi.nlm.nih.gov/protein/497225001?report=genbank&log$=prottop&blast_rank=2&RID=DMVNEZ5M013) | 2.00E-40 | 49% | PRK13258/PRK13258 | 7-cyano-7-deazaguanine reductase |
| 85 | + | 57431 | 57628 | None | n/a | n/a | n/a |  | Hypothetical protein |
| 86 | + | 57631 | 58923 | Enterobacteria phage 9g | [YP_009032326.1](https://www.ncbi.nlm.nih.gov/protein/640885076?report=genbank&log$=prottop&blast_rank=8&RID=DMVV80U3016) | 9.00E-05 | 27% |  | Queuosine tRNA-ribosyltransferase |
| 87 | + | 58923 | 59234 | None | n/a | n/a | n/a |  | Hypothetical protein |
| 88 | + | 59215 | 59550 | Desulfobacter postgatei | [WP_004075700.1](https://www.ncbi.nlm.nih.gov/protein/490177071?report=genbank&log$=prottop&blast_rank=2&RID=DMVY97TG016) | 2.00E-13 | 36% | Acetyltransf_1/pfam00583 | N-acetyltransferase |
| 89 | + | 59550 | 60758 | Rhizobium sp. 9140 | [CZT35567.1](https://www.ncbi.nlm.nih.gov/protein/1008908426?report=genbank&log$=prottop&blast_rank=2&RID=DMW019W3016) | 4.00E-95 | 44% | recD_rel/TIGR01448 | Exodeoxyribonuclease-5 |
| 90 | + | 60784 | 61122 | Xanthomonas fuscans subsp. fuscans | [KKY07586.1](https://www.ncbi.nlm.nih.gov/protein/821048594?report=genbank&log$=prottop&blast_rank=1&RID=DMW3FR3N016) | 6.00E-24 | 47% |  | Hypothetical protein |
| 91 | + | 61115 | 61552 | Oceanicola sp. S124 | [WP_010137344.1](https://www.ncbi.nlm.nih.gov/protein/497823188?report=genbank&log$=prottop&blast_rank=1&RID=DMW5ADT8013) | 4.00E-23 | 41% | DUF550/pfam04447 | Hypothetical protein |
| 92 | + | 61545 | 62213 | Marinifilum fragile | [WP_054722932.1](https://www.ncbi.nlm.nih.gov/protein/938942874?report=genbank&log$=prottop&blast_rank=1&RID=DMW7K6UJ013) | 3.00E-26 | 36% | DEDDh/cd06127 | DNA polymerase III, subunit epsilon |
| 93 | + | 62217 | 63191 | None | n/a | n/a | n/a |  | Hypothetical protein |
| 94 | + | 63206 | 63598 | None | n/a | n/a | n/a |  | Hypothetical protein |
| 95 | + | 63598 | 64431 | Spotted fever group | [WP_014408857.1](https://www.ncbi.nlm.nih.gov/protein/504221755?report=genbank&log$=prottop&blast_rank=22&RID=DMW9F341016) | 3.00E-29 | 31% | NrdG/COG0602 | Radical SAM protein |
| 96 | + | 64432 | 66447 | [Vibrio phage VpKK5](https://blast.ncbi.nlm.nih.gov/Blast.cgi#alnHdr_764162223) | [YP_009126593.1](https://www.ncbi.nlm.nih.gov/protein/764162223?report=genbank&log$=prottop&blast_rank=3&RID=DMWDD374013) | 3.00E-132 | 39% | PolA/COG0749 | Putative DNA polymerase A |
| 97 | + | 66444 | 66701 | None | n/a | n/a | n/a |  | Hypothetical protein |
| 98 | + | 66676 | 67140 | Bacteria symbiont BFo1 of Frankliniella occidentalis | [KMV67657.1](https://www.ncbi.nlm.nih.gov/protein/887505687?report=genbank&log$=prottop&blast_rank=3&RID=DMWH2B4P013) | 2.00E-29 | 41% | HNH_3/pfam13392 | HNH endonuclease |
| 99 | + | 67137 | 68057 | [Spirochaetes bacterium ADurb.Bin001](https://blast.ncbi.nlm.nih.gov/Blast.cgi#alnHdr_1167472893) | [OQC75326.1](https://www.ncbi.nlm.nih.gov/protein/1167472893?report=genbank&log$=prottop&blast_rank=1&RID=DMWKZWH6016) | 8.00E-50 | 35% | dnaX_nterm/TIGR02397 | DNA polymerase III, subunit gamma and tau |
| 100 | + | 68057 | 68503 | Sagittula stellata | [WP_005856452.1](https://www.ncbi.nlm.nih.gov/protein/492472784?report=genbank&log$=prottop&blast_rank=2&RID=DMWRNG3W016) | 1.00E-42 | 53% | deoxycytidylate_deaminase/cd01286 | Deoxycytidylate deaminase |
| 101 | + | 68496 | 68759 | None | n/a | n/a | n/a |  | Hypothetical protein |
| 102 | + | 68853 | 69134 | None | n/a | n/a | n/a |  | Hypothetical protein |
| 103 | + | 69158 | 69745 | Sphingomonadales | [WP_011950391.1](https://www.ncbi.nlm.nih.gov/protein/500508110?report=genbank&log$=prottop&blast_rank=5&RID=DMWUHXSF016) | 5.00E-52 | 48% |  | Transcription elongation protein SprT |
| 104 | + | 69827 | 70396 | None | n/a | n/a | n/a |  | Hypothetical protein |
| 105 | + | 70383 | 70862 | None | n/a | n/a | n/a |  | Hypothetical protein |
| 106 | + | 70862 | 71260 | None | n/a | n/a | n/a |  | Hypothetical protein |
| 107 | + | 71262 | 71393 | None | n/a | n/a | n/a |  | Hypothetical protein |
| 108 | + | 71380 | 71610 | None | n/a | n/a | n/a |  | Hypothetical protein |
| 109 | + | 71669 | 73306 | Bradyrhizobium ottawaense | [SDK44802.1](https://www.ncbi.nlm.nih.gov/protein/1085658819?report=genbank&log$=prottop&blast_rank=5&RID=DMWXAYAD013) | 9.00E-116 | 42% | UvrD/COG0210 | DNA or RNA helicase |
| 110 | + | 73309 | 73512 | Gluconobacter frateurii | [WP_048843751.1](https://www.ncbi.nlm.nih.gov/protein/889766217?report=genbank&log$=prottop&blast_rank=1&RID=DMXAMN8X013) | 3.00E-05 | 41% |  | Hypothetical protein |
| 111 | + | 73505 | 73720 | None | n/a | n/a | n/a |  | Hypothetical protein |
| 112 | + | 73717 | 73884 | None | n/a | n/a | n/a |  | Hypothetical protein |
| 113 | + | 73896 | 74534 | Rhizobium tropici | [WP_052227537.1](https://www.ncbi.nlm.nih.gov/protein/917681045?report=genbank&log$=prottop&blast_rank=1&RID=DMXDXSVE013) | 9.00E-22 | 34% |  | Hypothetical protein |
| 114 | + | 74531 | 75106 | Hafnia alvei | [WP_004091350.1](https://www.ncbi.nlm.nih.gov/protein/490192769?report=genbank&log$=prottop&blast_rank=3&RID=DMXG3U99013) | 2.00E-18 | 32% | DUF5051/pfam16473 | Exodeoxyribonuclease VIII |
| 115 | + | 75099 | 75422 | None | n/a | n/a | n/a |  | Hypothetical protein |
| 116 | + | 75419 | 75688 | None | n/a | n/a | n/a |  | Hypothetical protein |
| 117 | + | 75681 | 75887 | None | n/a | n/a | n/a |  | Hypothetical protein |
| 118 | + | 75884 | 76198 | None | n/a | n/a | n/a |  | Hypothetical protein |
| 119 | + | 76198 | 76446 | Brucella abortus LMN1 | [KFH18610.1](https://www.ncbi.nlm.nih.gov/protein/672581655?report=genbank&log$=prottop&blast_rank=1&RID=DMXGFEF0016) | 1.00E-12 | 46% |  | Hypothetical protein |
| 120 | + | 76443 | 76886 | None | n/a | n/a | n/a |  | Hypothetical protein |
| 121 | + | 76886 | 77215 | None | n/a | n/a | n/a |  | Hypothetical protein |
| 122 | + | 77261 | 77605 | None | n/a | n/a | n/a |  | Hypothetical protein |
| 123 | + | 77589 | 77861 | None | n/a | n/a | n/a |  | Hypothetical protein |

© 2017 by the authors; licensee MDPI, Basel, Switzerland. This article is an open access article distributed under the terms and conditions of the Creative Commons by Attribution (CC-BY) license (http://creativecommons.org/licenses/by/4.0/).
